# Turning Stem Cells Bad: Generation of Clinically Relevant Models of Human Acute Myeloid Leukemia through Gene Delivery- or Genome Editing-Based Approaches

**DOI:** 10.3390/molecules23082060

**Published:** 2018-08-17

**Authors:** Maria Mesuraca, Nicola Amodio, Emanuela Chiarella, Stefania Scicchitano, Annamaria Aloisio, Bruna Codispoti, Valeria Lucchino, Ylenia Montalcini, Heather M. Bond, Giovanni Morrone

**Affiliations:** 1 Laboratory of Molecular Haematopoiesis and Stem Cell Biology, Department of Experimental and Clinical Medicine, University Magna Græcia, 88100 Catanzaro, Italy; mes@unicz.it (M.M.); emanuelachiarella@unicz.it (E.C.); scicchitano@unicz.it (S.S.); aloisio@unicz.it (A.A.); valeria.lucchino@dzne.de (V.L.); ylenia.montalcini@studenti.unicz.it (Y.M.); 2 Laboratory of Medical Oncology, Department of Experimental and Clinical Medicine, University Magna Græcia, 88100 Catanzaro, Italy; amodio@unicz.it; 3 Tecnologica Research Institute-Marrelli Hospital, 88900 Crotone, Italy; bruna.codispoti@tecnologicasrl.com; 4 German Center for Neurodegenerative Diseases (DZNE), 53127 Bonn, Germany

**Keywords:** acute myeloid leukemia, chromosomal translocations, genetic aberrations, genome editing, leukemia stem cells, viral vectors, xenotransplants

## Abstract

Acute myeloid leukemia (AML), the most common acute leukemia in the adult, is believed to arise as a consequence of multiple molecular events that confer on primitive hematopoietic progenitors unlimited self-renewal potential and cause defective differentiation. A number of genetic aberrations, among which a variety of gene fusions, have been implicated in the development of a transformed phenotype through the generation of dysfunctional molecules that disrupt key regulatory mechanisms controlling survival, proliferation, and differentiation in normal stem and progenitor cells. Such genetic aberrations can be recreated experimentally to a large extent, to render normal hematopoietic stem cells “bad”, analogous to the leukemic stem cells. Here, we wish to provide a brief outline of the complementary experimental approaches, largely based on gene delivery and more recently on gene editing, employed over the last two decades to gain insights into the molecular mechanisms underlying AML development and progression and on the prospects that their applications offer for the discovery and validation of innovative therapies.

## 1. Introduction

Acute Myeloid Leukemia (AML) is a highly heterogeneous disease from a biological and molecular point of view, whose long-term prognosis remains dismal despite the considerable progress in therapeutic strategies developed over the last decades [1]. AML is the prevalent acute leukemia in adults and is characterized by the accumulation of immature myeloid blasts with incomplete differentiation and extensive proliferative potential. According to the current consensus, AML derives from the occurrence of at least two distinct oncogenic hits, which involve factors implicated in the control of differentiation and/or proliferation, such as hemopoietin receptors and signal transduction effectors, transcription factors, and epigenetic modifiers that regulate the physiological development of the hematopoietic system and the homeostasis of its stem and progenitor cell compartment [2,3,4,5,6,7].

AMLs typically harbor gene aberrations, frequently represented by chromosomal translocations, that, in some cases, are a hallmark of a specific leukemia sub-type. These translocations generate fusion genes encoding chimeric molecules with inappropriate functions that are likely to be involved in the development and/or progression of the leukemia, by fundamentally altering the normal features of stem/early progenitor cells [8]. A number of such aberrations have been identified, and some have been linked to the disruption of physiological processes in the cells where they occurred, suggesting that the genes affected are essential regulators of normal hematopoiesis [9,10].

AMLs have been shown to contain a subpopulation of highly immature cells, referred to as leukemia stem cells (LSCs) or leukemia-initiating cells (L-ICs), that share several features with normal hematopoietic stem cells, including a quiescent status and resistance to a variety of therapeutic agents. These cells, unlike the bulk of the malignant cell population, are able to generate AMLs when transplanted in immunocompromised recipients [11,12,13]. The importance of LSCs is evident, as this sub-population represents a critical target for therapeutic approaches aimed at the complete eradication of the leukemia. Equally relevant is the concept of cell-of-origin, that is the cell in which the transforming event(s) occur: The identification of this cell indeed represents a possibility to gain valuable insights into the molecular mechanisms that trigger the leukemic transformation of normal hematopoietic cells [10,13].

## 2. Gene Delivery-Based Strategies for AML Modeling

The availability of methods, to permanently introduce exogenous genetic material into the genome of mammalian cells has provided an unprecedented opportunity for dissecting normal hematopoiesis and modeling leukemias. Initially, gene transfer approaches were mainly based on the use of recombinant retroviruses, which are able to integrate into the genome of the target cells and to drive high levels of expression of the oncogenic proteins encoded by the cDNA contained in the vector (reviewed in [14]). In order to facilitate the identification or selection of the cells that had incorporated the viral genome, such viruses were further modified with the introduction of the coding sequence for proteins that confer antibiotic resistance, or for reporter fluorescent proteins, and often by the addition of an internal ribosome entry site (IRES) sequence between the transgene and the selectable/reporter gene, to allow transcription of a bicistronic mRNA and thus the simultaneous expression of both genes in the target cell [15]. One limitation of retroviral vectors is that they are considerably more efficient in infecting actively cycling cells, whereas the most primitive hematopoietic progenitors are typically slowly dividing or quiescent. To circumvent this problem, HIV-derived self-inactivating lentiviral vectors were also employed for gene delivery in hematopoietic cells and were used with success owing to their ability to efficiently target stem and early progenitor cells (reviewed in [16]).

A variety of lentiviral vectors are currently available, in which the expression of the transgene and/or the reporter protein is directed by the following: (i) strong, universal promoters that yield stably elevated expression levels in diverse types of target cells; (ii) inducible promoters that give the possibility to achieve different levels of gene expression in response to different doses of specific stimulants [17], or (iii) tissue-specific promoters, which ensure that transgene expression is restricted to, or preferential in, specific types of target cells [18,19]. Lentiviral vectors containing dual promoters driving the transcription of a transgene and a reporter gene, respectively, have also been generated and proved highly efficient in transducing primitive human hematopoietic cells [20].

In seminal studies conducted in the early 2000s using retroviral-mediated gene transfer, the groups of J. Gary Gilliland, Guy Sauvageau, and Keith Humphries provided the first evidence of the transforming activity of leukemia-associated aberrant genes, including those encoding constitutively active variants of regulatory molecules, such as mutants of the tyrosine kinase receptor FLT3 bearing internal tandem duplications in the juxta-membrane region (FLT3-ITD) or the fusion proteins NUP98-HOXA9 or NUP98/HOXD13. These studies showed that the introduction in mouse bone marrow cells of these candidate oncogenes led to the development of lethal myeloproliferation or AML in mice [21,22,23,24]. These findings prompted other laboratories to attempt to confirm and extend these data and initiated one of the most exciting chapters in the history of the modern experimental hematopoiesis. Mulloy and collaborators [25,26] transduced human primary CD34^+^ primitive hematopoietic progenitors, either isolated from apheresis of cytokine-mobilized peripheral blood cells (mPB-CD34^+^) of from neonatal, umbilical cord blood (CB-CD34^+^), with retroviral vectors carrying the cDNA of the fusion gene AML1-ETO, generated by the t(8;21) translocation and characteristically present in a substantial fraction of the M2 subtype of AMLs. Their work showed that the enforced expression of AML1-ETO rendered the transduced cells capable of long-term survival and expansion, without loss of their differentiation potential, both in liquid, cytokine-driven cultures, and in co-culture with stromal cells. Subsequently, using human CB-CD34^+^ cells, Chung et al. [27] and Schuringa et al. [28] demonstrated that the retroviral-mediated enforced expression of FLT3-ITD, as well as that of a constitutively active form of its downstream target STAT5a, resulted in the expansion of highly immature hematopoietic stem/progenitor cells. In long-term stromal co-cultures, these cells gave rise to early cobblestone areas (clusters of round, phase-dark hematopoietic cells embedded in the stromal cell monolayer, derived from the proliferation of highly primitive hematopoietic progenitors) and demonstrated extensive capacity of self-renewal. Shortly thereafter, the same group [29,30] showed that the transduction of human CB-CD34^+^ cells with NUP98-HOXA9 significantly enhanced stem cell self-renewal in vitro and also in vivo, when the cells expressing the fusion oncogene were transplanted in immunodeficient (non-obese diabetic-severe combined immunodeficiency, NOD/SCID) mice.

Unexpectedly, in the long-term stromal co-cultures of CD34^+^ cells transduced with both FLT3-ITD and constitutively active STAT5a, a robust erythropoietic activity, typically absent in these types of co-cultures, was observed [27,28,31]. These findings nicely dovetailed with the data reported at the same time by Levine et al. [32], who detected in the great majority of myeloproliferative syndromes-and in particular in almost all cases of polycythemia vera-a V617F mutation occurring in the coding sequence of the Janus kinase 2 (JAK2) that constitutively activated this receptor-associated tyrosine kinase, resulting in turn in the strong activation of STAT5. Subsequent investigations confirmed the results of these studies and provided additional insights into the biological effects and the mechanism of action of constitutively-active FLT3-ITD and STAT5a, as well as of the NUP98-HOXA9 fusion protein in normal and malignant hematopoietic stem and progenitor cells [33,34,35,36,37,38,39,40].

## 3. Mixed Lineage Leukemia (MLL) as a Versatile Tool to Dissect AML

A significant breakthrough in the in vitro and in vivo modeling of human AMLs came from the demonstration, by John Dick’s group, that transduction with retroviruses containing MLL-derived fusion oncogenes could fully transform human early hematopoietic progenitors and render them leukemogenic in immunocompromised mice [41].

The MLL gene, located on chr. 11q23.3, encodes the histone-lysine N-methyltransferase 2 (KMT2A) protein, also referred to as ALL-1 or MLL. This protein, through its interaction with a complex network of epigenetic modifiers, plays a key role in the development of the hematopoietic system. Among the better-known activities of KMT2A is the regulation of the Hox gene cluster, which is implicated in the control of normal hematopoiesis and whose dysregulation is often associated with the development of leukemia [42,43,44]. The 11q23.3 region is extremely prone to translocations, which cause the in-frame fusion of MLL with almost 100 partner genes [45] and generate fusion oncoproteins associated with acute leukemias, generally characterized by poor prognosis, with lymphoid or myeloid phenotype, or both, hence the name mixed lineage leukemia (MLL), commonly used to designate this gene. The most frequent MLL-derived fusion oncogenes are MLL-AF4, MLL-ENL, and MLL-AF9, with the former being typically present in acute lymphoblastic leukemias, the latter prevalently in AMLs, and MLL-ENL associated with both ALLs and AMLs [45].

To elucidate the properties of MLL-AF9, Rabbitts and collaborators [46,47] generated an MLL-AF9 fusion gene in mouse ES cells by homologous recombination and showed that the expression of this gene, driven by the MLL promoter, resulted in the development of AMLs that led to death within 12 months. In a complementary approach, the same group set up a system to achieve recombination of MLL and ENL during mouse development. To this end, they introduced loxP sites into introns flanking the breakpoint of the relevant genes and crossed the mice thus obtained with mice carrying the Cre recombinase under the transcriptional control of the hematopoietic Lmo2 promoter [48]. The recombination between MLL and ENL resulted in the development of myeloid leukemias with early onset and high penetrance.

In an effort to identify the cell-of-origin of MLL-AF9-associated AMLs, Lavau et al., Cozzio et al., Krivtsov et al. [49,50,51], and Somervaille and Cleary [52] used retroviral vectors to transduce different murine hematopoietic progenitors with MLL fusion oncogenes and reported that both bona fide hematopoietic stem cells, as well as committed (common myeloid and granulo-monocytic) progenitors could be transformed by MLL-ELL, MLL-ENL, and MLL-AF9 into cells with leukemia-initiating properties.

These studies, however, presented potential problems due to the strong overexpression of the transgenes caused by multiple viral insertions in the genome of the target cells and/or by the potent transcriptional activity of the retroviral promoters. To circumvent these problems, Kersey and collaborators [53] analyzed MLL-AF9 knock-in mice, where the expression of the transgene was directed by the endogenous MLL promoter and hence expected to be “physiological” both in terms of levels and of cell-specificity. The results of this study revealed that the expression of MLL-AF9 driven by the endogenous MLL promoter, although much lower than that observed in retrovirally transduced cells, was significantly higher in stem cells than in committed myeloid progenitors and was far more effective in transforming stem cells and common lymphoid progenitors compared with common myeloid and granulo-monocytic progenitors.

Regarding the human system, as mentioned above, the study of Barabé et al. [41] represented a highly significant step forward, in that it provided the first conclusive evidence that AML-associated oncogenes derived from MLL rearrangements; in this case, MLL-ENL and MLL-AF9-could transform human primitive hematopoietic cells as a single hit and confer on them leukemogenic properties. One unexpected and somewhat puzzling finding in this study, however, was that human cells transduced with MLL-ENL—that is equally present in MLL-associated ALLs and AMLs—gave rise almost invariably to B-ALL when transplanted into immunocompromised (NOD/SCID) mice. This appeared in contrast with the behavior of mouse bone marrow cells transformed with the same oncogene, which had been found to generate predominantly AML when transplanted in syngeneic mice [54]. Furthermore, in the Barabè study [41], CB-CD34^+^ cells transduced with MLL-AF9, typically associated with AMLs, induced the development of AMLs but also B-ALLs, as well as acute biphenotypic leukemias (ABL) in NOD/SCID mice. These apparent inconsistencies raised the question of whether the activity of MLL-fusion protein was different in human versus mouse cells, or if the difference might be due to differential effects of the human and mouse microenvironment. Indeed, it is known that cytokines that are critical inducers of myelopoiesis, such as interleukin-3 (IL-3), as well as the granulocyte-macrophage colony-stimulating factor (GM-CSF), and are strictly species-specific in their activity, and therefore, the mouse bone marrow stroma does not support with optimal efficiency human myelopoiesis.

This issue was addressed in an elegant set of experiments by J. Mulloy and collaborators [55], who analyzed the biological properties of MLL-AF9-transduced human CB-CD34^+^ cells in vitro and in vivo. In vitro, these cells became immortalized and displayed unlimited proliferation potential and myeloid immuno-phenotypic features. However, when subjected to culture conditions that promote B-cell growth, the MLL-AF9-transformed cells exhibited a mixed-lineage phenotype predominantly represented by immature B-cells. When transplanted in NOD/SCID (NS) or NOD/SCID-β2microglobulin^−/−^(NS-B2M) immunodeficient mice directly after transduction, MLL-AF9-CD34^+^ cells gave rise to AML, B-ALL, and ABL. If the same cells, however, were injected in NS recipients transgenic for the genes encoding human stem cell factor (SCF), IL-3, and GM-CSF, they consistently generated AMLs, indicating that microenvironmental signals play a role of pivotal importance in the lineage determination of MLL-transformed leukemia stem cells [55].

In addition to environmental signals, however, the cell-intrinsic properties of the cells-of-origin also exert a critical influence on the outcome of MLL-AF9-mediated transformation of human primitive hematopoietic progenitors. This was demonstrated by Horton et al. [56], who performed lentiviral-mediated transduction of neonatal (CB-derived) or adult (bone marrow-derived) CD34^+^ cells. These experiments showed that, while in the former population, MLL-AF9 efficiently immortalized cells of both myeloid and lymphoid lineage, and in adult cells, the immortalization was significantly less efficient and strongly myeloid-biased. These findings are consistent with the fact that pediatric acute leukemias harboring the MLL-AF9 rearrangement often display mixed lineage features, whereas their adult counterpart is predominantly represented by AMLs. This also fits well with the notion that myeloid-biased hematopoietic stem cells are significantly more abundant in the adult than in the fetus and/or neonate, where instead a “balanced” lympho-myeloid stem cell population is more highly represented [57].

Taken together, the results of the studies briefly illustrated above indicate that MLL fusion oncogenes represent a useful and versatile tool to investigate the mechanisms leading to AML development and to search for potential therapeutic targets. These and further investigations led to the identification of several factors that cooperate with MLL-AF9 and are critically required for its leukemogenic activity. Among these are the receptor tyrosine kinase FLT3 [55,56,58,59]; HoxA9, one of the prominent target genes of MLL [60,61]; the GTPase Rac1 [55,62,63]; a variety of epigenetic modulators including the H3K79 methyltransferase DOT1L [64,65,66,67], the AAA+ ATPase RUVBL2 [68], the histone 2B ubiquitin ligase, RNF20 [69], and the histone acetyltransferase KAT2A; the RNA-binding protein Musashi2 [70]; and the multi-zinc finger transcription co-factor, ZNF521 [71,72,73,74]. All these factors can be considered potential actionable targets for AMLs bearing MLL aberrations. It is important to notice that, for several of them, synthetic inhibitors are available and have been validated, and some of them are being tested in preclinical models or clinical trials [75,76,77,78,79,80].

Another class of important regulatory molecules, whose expression is influenced by fusion oncoproteins, are microRNAs [81]. Up- or downregulation of the expression of microRNAs, induced by MLL fusion proteins or by their downstream targets, such as HOX proteins, has been shown to contribute to the development of MLL-r AMLs; these molecules may represent additional attractive targets for therapeutic intervention [82,83,84].

MLL-AF9-transformed cells were also used to design and implement a sophisticated approach for the identification of novel agents suitable for targeting leukemia stem cells in the context of the stromal microenvironment, which is believed to play a key role in promoting the maintenance and expansion of this subpopulation of leukemic cells. This approach was based on the evidence that oncogene-transformed CD34^+^ cells, as well as putative AML stem cells, when co-cultured with bone stromal cell lines that support long-term hematopoiesis, generate cobblestone areas forming cells with extensive self-renewal potential [27,28,85,86]. In this project, carried out in collaboration by five laboratories at the Broad Institute and at Memorial Sloan-Kettering Cancer Center, an automated system was developed for the scoring of red cobblestone areas derived from MLL-transformed, leukemogenic mouse progenitor cells expressing the DS-Red fluorescent protein, in co-cultures with green fluorescent protein (GFP)-transduced OP-9 stromal cells. A high-throughput screening of over 14,000 synthetic compounds was conducted using these co-cultures, which led to the discovery of 155 molecules (including the sesquiterpene lactone, parthenolide, known for its potent cytotoxic activity on LSC, as well as inhibitors of the mevalonate pathway) capable of selectively abrogating the formation of leukemic but not normal, cobblestone areas in a context that, in several regards, recapitulates the interactions between leukemia stem cells and the bone marrow microenvironment [87,88]. Because MLL-AF9 readily immortalizes human CD34^+^ cells, it is presumable that similar screenings may be carried out in the future using human MLL-AF9-transformed primitive progenitors, in order to test the candidate therapeutic agents in a system more closely related to the pathophysiology of human AML.

## 4. Emerging Technologies and Systems

Novel technologies and tools, which have recently become available, may be amenable to the development of physiologically-relevant models for the dissection of AMLs and the search for, and/or validation of, new and effective therapeutic agents. Among these, some of the most interesting technologies are those for genome editing in mammalian cells.

These methods are based either on the use of recombinant nucleases (zinc-finger nucleases (ZNF) and transcription activator-like effector nucleases (TALEN)) in which DNA-binding motifs are engineered to specifically bind to the desired sequence(s) in the genome of the target cell and fused to the DNA-cleavage domain of the restriction enzyme, FokI [89], or on the recruitment to the target location in the genome, via a synthetic guide RNA, of the nuclease-designated CAS9 (clustered regularly interspaced short palindromic repeats (CRISPR)-associated protein 9) [90]. The activity of all the above nucleases introduces double-strand breaks at the target sites, which are repaired by the cell DNA repair machinery through mechanisms involving homologous recombination (HR) or non-homologous end joining (NHEJ), in a process that can be exploited to introduce, insertions, deletions, or substitutions in correspondence to the double-strand breaks [91].

These genome-editing strategies have been successfully employed to induce chromosomal translocation producing MLL-AF9 and MLL-ENL fusion genes under the control of the endogenous MLL promoter in human CB-CD34^+^ cells. The resulting cells did not display the full phenotype of transformed cells in vitro, in particular with regard to unlimited growth/self-renewal potential. However, when injected in immunodeficient mice, they were able to produce leukemias with features that closely resembled those of the leukemias driven by MLL rearrangements [92,93,94]. Based on this evidence, it may be envisioned that in the near future, genome-editing techniques may be exploited to produce arrays of endogenous gene fusions, even at the individual patient level, and/or to achieve the knock-in of multiple AML-associated aberrant genes, alone or in combination, to finely dissect the molecular mechanisms of myeloid leukemogenesis.

The problem remains, however, to identify a suitable and physiologically relevant host for the leukemogenic cells generated with genome editing, especially considering that immunodeficient mice typically show a limited capacity to promote the engraftment of human leukemia-initiating cells. In addition, as discussed above, the ability of these animals to support human hematopoiesis is generally lymphoid-biased or, when the mice are rendered transgenic for human myeloid hemopoietins, significantly myeloid-biased. In this regard, a recently-developed model originally set up in the laboratory of P. Tassone [95] and subsequently refined by the groups of JJ. Schuringa [96,97] and R. Majeti [98], may prove to be of considerable value. In this model, synthetic scaffolds are coated with primary human bone marrow-derived mesenchymal stem cells, capable of differentiating into the major cellular components of bone marrow stroma, and implanted subcutaneously in severely immunocompromised NOD/SCID-IL2R gamma chain knock-out (NSG) mice. When oncogene-transformed CD34^+^ cells or primary human myeloid leukemic cells are injected in these implants containing humanized hematopoietic niche, they readily engraft with high efficiency and give rise to serially-transplantable leukemias that recapitulate, to a large extent, the original disease.

## 5. Conclusions and Perspectives

In the last two decades, the development of novel technologies and tools to manipulate gene expression in primitive progenitors of the hematopoietic system, has enabled researchers to “turn stem cells bad” as summarized in Table 1. The resulting, considerable progress in our knowledge of the mechanisms and molecules implicated in myeloid leukemogenesis has allowed the generation of useful preclinical models of AML and led to the identification of molecular targets and of novel agents with therapeutic potential, several of which are already approved, or are currently been tested, for clinical use. It can be predicted that the application of genome-editing techniques for the production, in normal human hematopoietic stem/progenitor cells, of endogenous genetic aberrations faithfully resembling those observed in AMLs, together with the availability of recipient animals, which harbor a humanized bone marrow niche, may be exploited to establish preclinical models that even more realistically reflect the pathophysiological scenario of AMLs, with particular regard to the LSC-niche interaction. This will represent an invaluable tool for the discovery and validation of novel therapeutics for combating acute myeloid leukemia through the effective eradication of the leukemia-initiating cell population.

## Figures and Tables

**Table 1:** Overview of principal experimental studies cited in this review.

Gene	Methodology	Vector	Species	Cellular Target	Phenotype	Ref.
AML1-ETO	Gene Transfer	Retro	H	MPBC-CD34^+^	Self-renewal	[25]
FLT3-ITD	Gene Transfer	Retro	m	BM-PC	Myeloproliferation	[22]
		Retro	H	CB-CD34^+^	Self-renewal/Erythropoiesis	[27]
HOXA9	Gene Transfer	Lenti	m	CMPs	AML	[60]
MLL-AF9	Homologous recombination		m		AML	[46]
	Knock-in		m		Myeloproliferation/AML	[47]
					AML	[53]
	Gene Transfer	Retro	m	BM-MNCs	AML	[59,67]
		Retro	H	CB-CD34^+^	Transformation (Lymphoid/Myeloid)	[55,63]
		Retro	m	CMPs	AML	[51]
		Lenti	H	BM-CD34^+^/CB-CD34^+^	Transformation (Lymphoid/Myeloid)	[56]
				CB-CD34^+^	Transformation (Lymphoid/Myeloid)	[63]
			H/m	Lin^−^ BM cells/CB-CD34^+^	Leukemia	[68]
			m	CMPs	AML	[51]
	TALEN		H	CB-CD34^+^	Transformation, acute leukemia	[93]
MLL-ENL	CRISPR-Cas9		H	CB-CD34^+^	Proliferation, acute leukemia	[94]
	Gene Transfer	Retro	H	CB-Lin^−^	AML/ALL	[41]
				BM-MNCs	AML	[48]
	TALEN		H	CB-CD34^+^	Transformation, acute leukemia	[93]
MLL-ELL	Gene Transfer	Retro	m	Lin^−^ BM cells	AML	[49]
NUP98/HOXA9	Gene Transfer	Retro	H	BM-MNCs	Self-renewal	[21]
			m	BM-MNCs	CML blast crisis	[23]
			H	CB-CD34^+^	Self-renewal	[29]
	Transgenic		Z			[38]
	Transgenic		D			[40]
NUP98/HOXD13	Gene Transfer	Retro	m	BM-MNCs	Self-renewal/Myeloproliferation	[24]
STAT5a	Gene Transfer	Retro	H	CB-CD34^+^	Self-renewal/Erythropoiesis	[28,31,35,36]
			m	FL-PCs	Erythropoiesis	[34]

**Abbreviations: H:** man: **m:** mouse; **Z:** zebrafish; **D:** Drosophila; **MPBC-CD34^+^:** peripheral blood mobilized CD34^+^ cells; **CMP:** common myeloid progenitors; **FL-PCs:** fetal liver-derived hematopoietic progenitors; **BM-MNCs:** Bone marrow-derived mononuclear cells; **CB-CD34^+^:** Umbilical cord blood derived CD34^+^ stem cells; **BM-CD34^+^:** Bone marrow-derived CD34^+^ cells; **Lin^−^:** lineage marker depleted.
